# Exploring the Thermal Stability of Sb_2_Se_3_ for Potential Applications through Advanced Thermal Analysis Methods

**DOI:** 10.1021/acsomega.4c10053

**Published:** 2025-05-26

**Authors:** Gozde Altuntas, Mehmet Isik, Gokhan Surucu, Mehmet Parlak, Ozge Surucu

**Affiliations:** a Faculty of Technology, Department of Metallurgical and Materials Engineering, Gazi University, Ankara 06500, Turkey; b Department of Biomedical Engineering, Faculty of Engineering and Architecture, Izmir Bakircay University, Izmir 35665, Turkey; c Biomedical Technologies Design Application and Research Center, Izmir Bakircay University, Izmir 35665, Turkey; d Faculty of Technology, Department of Energy Systems Engineering, Gazi University, Ankara 06500, Turkey; e Department of Physics, Middle East Technical University, Ankara 06800, Turkey; f Department of Electrical and Electronics Engineering, Atilim University, Ankara 06836, Turkey

## Abstract

Antimony selenide (Sb_2_Se_3_) is a promising material for energy applications, including photovoltaics, thermoelectrics, and photodetectors, due to its favorable electronic properties, availability, and low toxicity. However, its thermal stability, crucial for device efficiency and reliability, has been less explored, leaving a gap in understanding its high-temperature suitability. This study evaluates the thermal stability of Sb_2_Se_3_ using thermogravimetric analysis (TGA), differential thermal analysis (DTA), and differential scanning calorimetry (DSC). The results show that Sb_2_Se_3_ remains stable up to 500 °C, with two significant weight loss stages: 1.75% between 500 and 610 °C, and 3.50% between 610 and 775 °C, indicating decomposition processes. Activation energies for the decomposition phases were determined as 121.8 and 57.2 kJ/mol using the Coats–Redfern method. Additionally, an endothermic phase transition was observed between 599 and 630.6 °C via DSC analysis. These findings demonstrate Sb_2_Se_3_’s potential for high-temperature energy applications, providing essential insights for optimizing its use in solar cells, thermoelectric devices, and other technologies.

## Graphical abstract

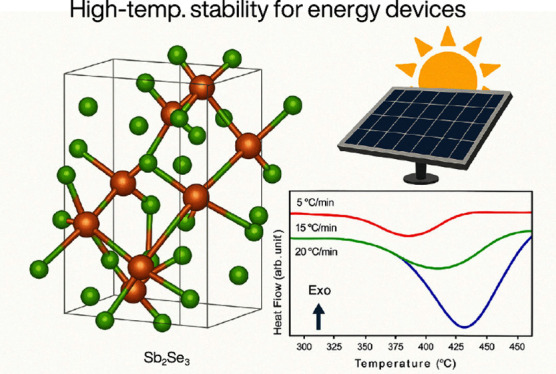

## 1 Introduction

The exploration of efficient and cost-effective materials for energy applications has attracted significant attention in recent years, fueled by the pressing demand for sustainable and renewable energy solutions.^1–4^ The quest for novel semiconductor materials has led to an increasing focus on compounds that can balance performance, cost, and environmental impact. Antimony selenide (Sb_2_Se_3_) has emerged as a promising candidate due to its favorable electronic properties, abundant availability, and low toxicity, making it attractive for a variety of applications, including photovoltaics, thermoelectrics, and photodetectors.^2,3,5–8^ The distinctive crystal structure of Sb_2_Se_3_ contributes to its potential, offering desirable optical and electronic properties.^3,6–8^ However, understanding the thermal stability of Sb_2_Se_3_ is crucial, as its performance in real-world applications heavily depends on its ability to withstand varying thermal environments.

Thermal stability analysis provides critical insights into the material’s behavior under heat, helping researchers determine the conditions under which the material remains chemically and structurally intact.^9–11^ This aspect is particularly important for devices that experience fluctuating temperatures, such as solar cells and thermoelectric modules, where temperature variations can lead to changes in material properties, affecting efficiency, reliability, and overall device performance. Ensuring that Sb_2_Se_3_ remains stable under these conditions is essential for maintaining consistent output and prolonging the operational lifetime of these devices. Despite its promising optoelectronic properties, there has been limited focus on the thermal behavior of Sb_2_Se_3_, which creates a significant knowledge gap that needs to be addressed for its practical application.

In this study, an in-depth analysis of the thermal stability of Sb_2_Se_3_ was conducted using advanced thermal analysis techniques, including TGA, DTA, and DSC. The findings demonstrated that Sb_2_Se_3_ exhibited considerable thermal stability up to approximately 500 °C, with significant weight loss occurring only at higher temperatures, indicative of decomposition. The activation energies of the decomposition processes were determined using the Coats–Redfern method, providing valuable insights into the thermal behavior of the material. Furthermore, DSC analysis revealed an endothermic phase transition, further highlighting the stability characteristics of Sb_2_Se_3_. These results contribute to a comprehensive understanding of its thermal properties, which is crucial for optimizing its use in high-temperature applications, such as solar cells and thermoelectric devices. This study filled an important gap in the literature, providing a foundation for the future development and practical deployment of Sb_2_Se_3_ in energy applications.

## 2 Experimental Details

The crystal growth of antimony selenide (Sb_2_Se_3_) using the Bridgman method involved the use of high-purity elemental precursors of selenium (Se) and antimony (Sb). For this study, the selenium precursor used was 99.99% pure and sourced from Sigma-Aldrich, while the antimony precursor was 99.999% pure and sourced from Thermo Scientific. The stoichiometric amounts of these precursors, calculated to synthesize 15 g of Sb_2_Se_3_, included approximately 7.59 g of Sb and 7.41 g of Se. The antimony powder had a particle size of −200 mesh, ensuring appropriate reaction kinetics and homogeneity. The Bridgman technique is a well-established method for growing high-quality crystals, particularly for materials with applications in semiconductors and optoelectronics. To begin the process, the high-purity selenium and antimony were precisely weighed and loaded into a clean quartz ampule. The ampule, measuring 15–20 cm in length, was designed to withstand high temperatures and maintain an inert atmosphere during the entire growth process. It was then evacuated to a pressure of about 10^–5^ Torr to remove any residual gases, which could lead to unwanted reactions and impurities in the crystal. Once evacuated, the ampule was sealed under vacuum using a torch. The sealed ampule containing the Sb and Se precursors is then placed vertically in a Bridgman furnace, which had a temperature gradient along its length. The upper portion was maintained at a higher temperature to ensure complete melting of the materials, while the lower portion was kept at a lower temperature to facilitate controlled crystallization. The furnace was heated to a temperature of around 650–750 °C, exceeding the melting point of Sb_2_Se_3_, to ensure that both elements fully react and melt, forming a homogeneous molten solution. The ampule was then slowly lowered through the temperature gradient in the furnace at a rate of 0.5–1.0 mm per hour. This slow cooling and solidification process allowed for the controlled crystallization of Sb_2_Se_3_, promoting the growth of large, defect-free crystals. The resulting crystals were observed to exhibit a distinct layered structure, as confirmed by SEM imaging. Nucleation began at the cooler end of the ampule and progressed upward, resulting in the formation of a predominantly single-crystal material, under the controlled conditions utilized in this study. Once the crystal growth process was complete, the ampule was gradually cooled to room temperature to avoid thermal shock, which could compromise the structural integrity of the crystal. The quartz ampule was then carefully broken to extract the grown Sb_2_Se_3_ crystal. The extracted crystals were typically dark gray to black in color.

The grown Sb_2_Se_3_ crystals were further characterized using various techniques. For crystallographic analyses, X-ray diffraction (XRD) was performed using a Bruker D8 Advance brand X-ray Diffractometer at a scanning speed of 0.05°/min and a diffraction angle range of 20–60°. Cu Kα (λ = 1.54056 Å) was used as the X-ray source, and the patterns were analyzed at 40 kV and 40 Ma operating conditions. Microstructural examinations were performed using a scanning electron microscope (SEM) Hitachi SU8700. Elemental mappings were analyzed with the energy-dispersive spectroscopy (EDS) detector connected to this device. TGA and DTA experiments were performed on a Hitachi STA7300 simultaneous device at a heating rate of 10 °C/min in an argon atmosphere at an operating temperature of 25–800 °C. Differential scanning calorimetry (DSC) experiments were performed using a HITACHI DSC 7020 thermal analysis unit. Tests were performed in the temperature range of 300 to 450 °C in an argon atmosphere using heating rates of 5, 15, and 20 °C/min. Each test sample weighing 8 mg was placed in aluminum pans for analysis.

## 3 Results and Discussion

XRD measurements were conducted to investigate the crystalline structure of the Sb_2_Se_3_ compound. The resulting XRD spectrum, recorded in the 20–60° range, is presented in Figure 1. As seen in the figure, 20 well-defined peaks were observed in the XRD pattern. These peaks were compared with the results of studies in the literature and with the data on standard cards.^12–14^ As a result of the comparisons, it was revealed that the observed peaks belonged to the Sb_2_Se_3_ material with orthorhombic structure. Moreover, the experimental diffraction pattern was analyzed through Rietveld refinement using the MAUD software. The calculated diffraction pattern, represented by the red curve, exhibits excellent consistency with the experimental data, validating the accuracy of the structural model. The refinement results determined the lattice parameter of the orthorhombic unit cell to be *a* = 11.770 Å, *b* = 3.9757 Å, and *c* = 11.6327 Å. Based on the analysis result, the observed peaks correspond to the Miller indices given on them in Figure 1. The Rietveld analysis resulted in *R*_wp_ value of 32.6%, which shows a good match between the experimental and calculated patterns. The *R*_b_ value of 25.1% also indicates a reasonable agreement in peak intensities. The χ^2^ value of 2.68 suggests a decent fit but leaves room for improvement.

**Figure 1:**
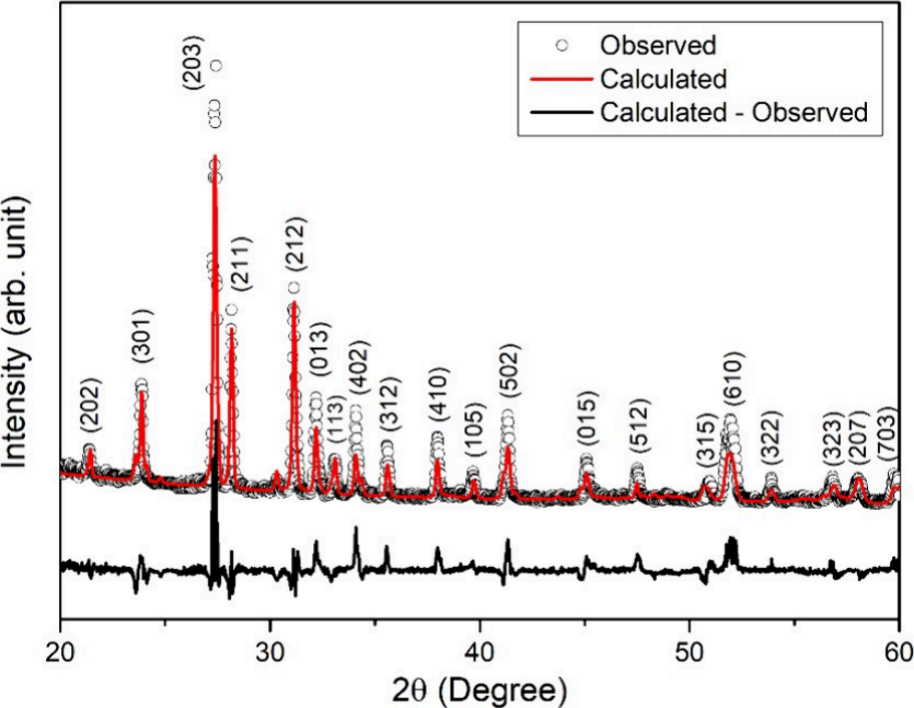
XRD pattern of the Sb_2_Se_3_ powder.

Williamson and Hall proposed that the broadening observed in diffraction peaks is due to combined effects from both crystallite size and strain within the crystal lattice. The Williamson–Hall Equation is formulated as^15,16^

$$\beta_{hkl}=\beta_{d}+\beta_{\varepsilon }$$ 1

where β_*hkl*_ is the FWHM of the diffraction peak corrected for instrumental broadening, β_d_ is the broadening due to crystallite size, and β_ε_ is the broadening due to strain effects. The following expressions are used to calculate crystallite size broadening and strain induced broadening

$$\beta_{d}=0.94\lambda /D\cos\theta$$ 2

$$\beta_{\varepsilon }=4\varepsilon \tan\theta$$ 3

where *D* is the average particle size, λ = 0.15406 nm, and ε is the root-mean-square of the microstrain. When these two relations are placed into eq 1, β_*hkl*_ is given as follows.^15,16^

$$\beta_{hkl}=\beta_{d}+\beta_{\varepsilon }=\frac{0.94\lambda }{D\cos\theta }+4\varepsilon \tan\theta$$ 4

A plot of βcosθ against 4sinθ, based on this equation, yields a slope representing the strain and *y*-intercept equivalent to *k*λ/*D*. Figure 2 shows the corresponding plot obtained using the experimental data of the eight most intense XRD peaks. As a result of linear fit application, strain and crystalline size were found to be −1.8 × 10^–3^ and 31.7 nm, respectively.

**Figure 2:**
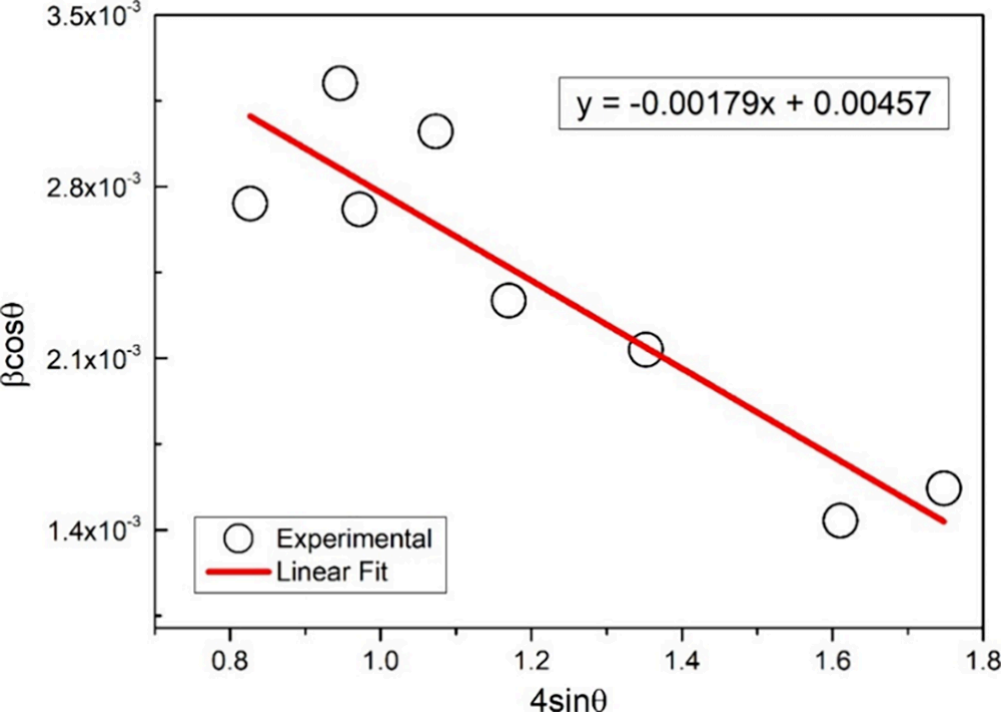
Williamson–Hall plot of βcosθ against 4sinθ. The solid line represents the linear fit.

Figure 3 indicates the elemental mapping and electron image of the Sb_2_Se_3_ crystal. In the top row and bottom row maps, Se (orange) and Sb (blue) elements appear to be distributed homogeneously, indicating that the component is well mixed and provides homogeneity of Sb_2_Se_3_ throughout the crystal. The distribution of both elements does not show significant differences between regions, indicating that the desired composition is achieved. The SEM image reveals that the structure of the crystal is layered, and it shows a fragmented or stacked structure in some places. Such structures may correspond to the layered crystal structure of Sb_2_Se_3_, and generally, these structures may influence electron transport or photovoltaic properties. The elemental composition of the Sb_2_Se_3_ crystal was examined using energy-dispersive X-ray spectroscopy (EDS). To determine the composition more reliably, EDS measurements were taken from different regions of the sample. The spectrum, as shown in Figure 4, confirms the presence of antimony (Sb) and selenium (Se) as the primary constituents. The atomic ratio was determined to be Sb: 37.3% and Se: 62.7%. These values correspond to the measurement closest to the average among the different regions analyzed. The variation in elemental ratios among these measurements was approximately ±2 at. %, and thus, the measurement uncertainty is considered to be ±2 at. %. Given this uncertainty, the obtained atomic ratios are consistent with the nominal Sb_2_Se_3_ composition. The absence of significant impurity peaks indicates the high purity of the sample, supporting its suitability for further thermal characterizations.

**Figure 3:**
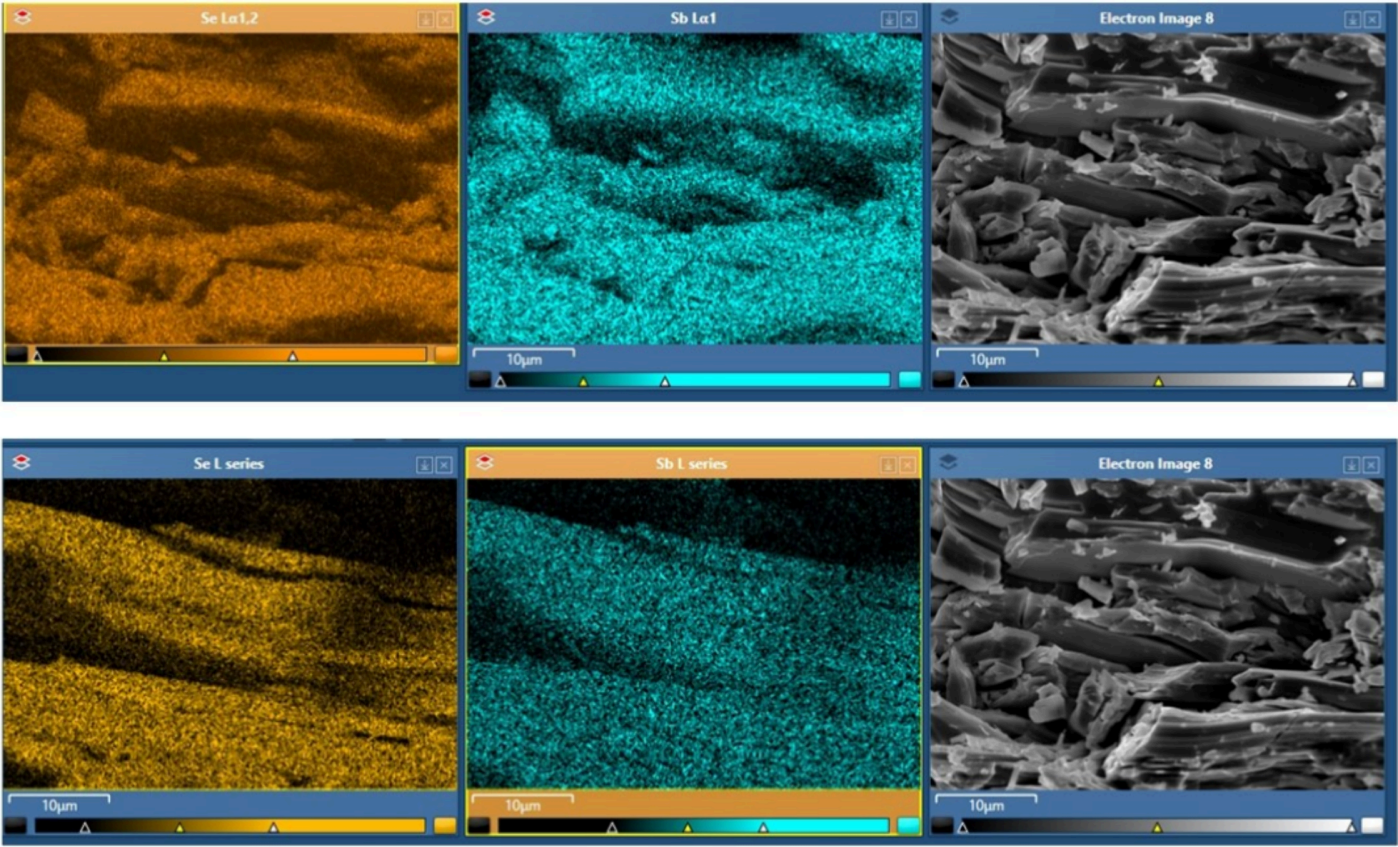
Elemental mapping of Sb and Se elements and electron image of the Sb_2_Se_3_ crystal.

**Figure 4:**
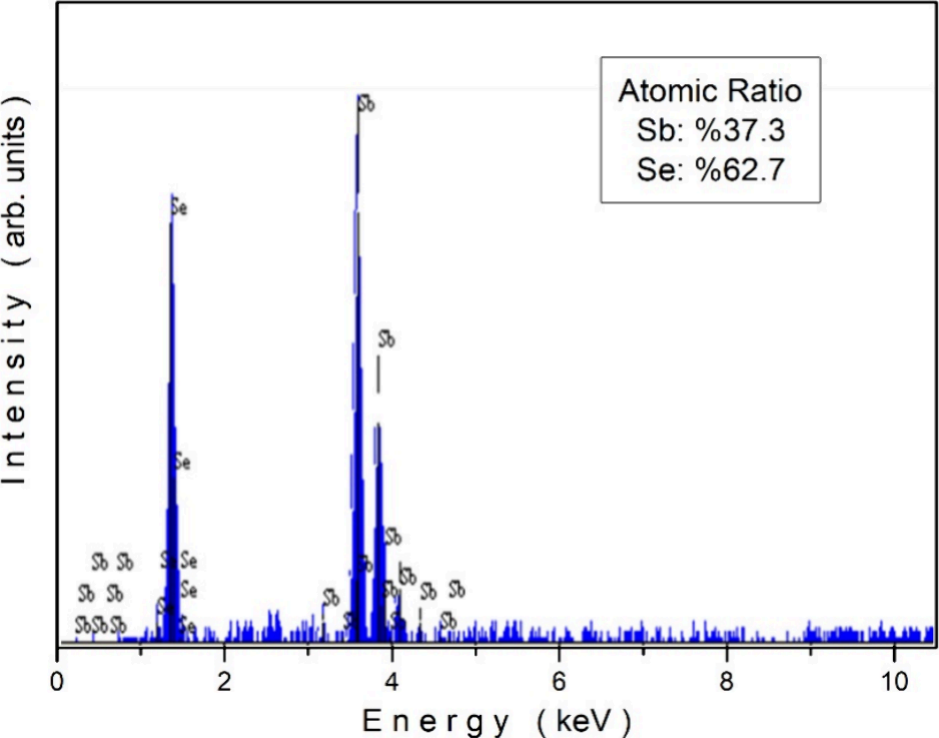
EDS spectrum of the Sb_2_Se_3_ crystal.

Figure 5 shows the TGA thermograms of the Sb_2_Se_3_ crystal in the 25–790 °C range. The basic features and possible interpretations we can extract from the graph are as follows: The TGA data indicated that the material remains stable up to 500 °C with no significant weight loss observed. A minor weight loss (∼0.1%) is detected around 300 °C; however, this change is within the experimental noise and does not influence the overall stability assessment or conclusions of the study. Little or no weight loss is observed in the region up to 500 °C, indicating that Sb_2_Se_3_ is quite stable in this temperature range. In the graph, two significant weight loss zones are observed. Δ*m*_1_ = 1.75%: The region that begins at approximately 500 °C and results in a weight loss of 1.75%. Δ*m*_2_ = 3.50%: A larger weight loss of 3.50% occurs between 610 and 775 °C. Although it exhibits a relatively stable behavior with small losses in mass up to about 610 °C, after this temperature, a significant mass loss begins. Above approximately 775 °C, a sharp weight loss is observed.

**Figure 5:**
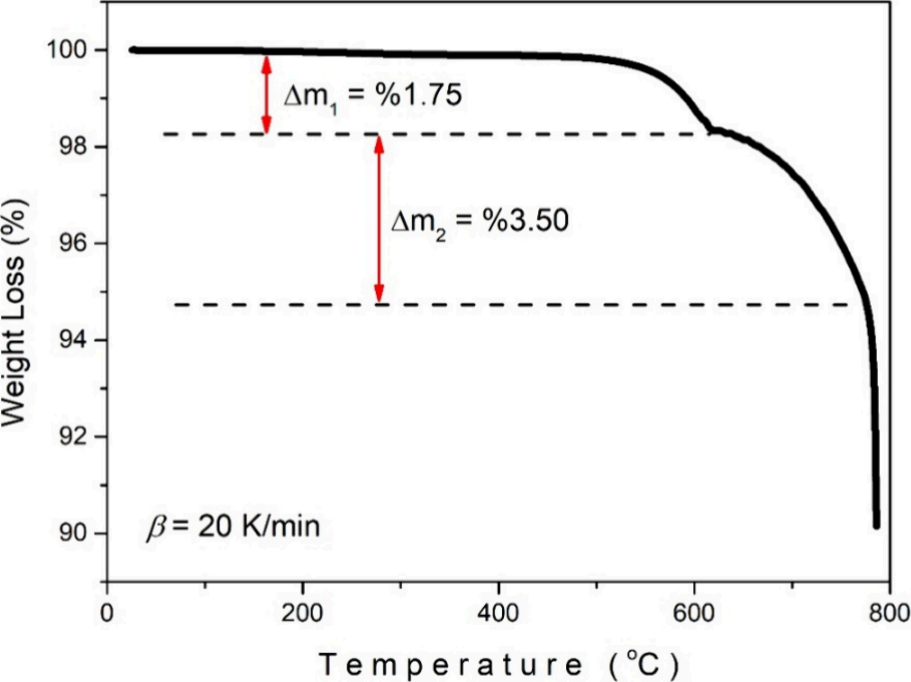
TGA curve of the Sb_2_Se_3_ crystal.

Thermal properties of Sb_2_Se_3_ were previously reported in the literature. The TGA plot of the Sb_2_Se_3_ powder indicated the presence of two weight loss regions.^17^ Weight loss started at around 423 °C and then sharply increased after 608 °C, which is the melting point of the compound. The evaporation behavior of the compound was also studied in refs 18,19 . Antimony (Sb) exhibits a relatively low vapor pressure compared to selenium (Se), which has a high vapor pressure, making selenium more prone to volatilization at elevated temperatures.^17^ The vapor pressure of Sb_2_Se_3_ lies between that of its constituents and increases significantly above 600 °C, facilitating the thermal evaporation of selenium and antimony species, as well as the formation of volatile decomposition products such as Sb_4_, SbSe, and Se_2_.^17^ These vapor pressure differences play a critical role in the observed weight loss during thermal analysis. Considering the results of these studies carried out on the Sb_2_Se_3_ material, the mass losses seen in our TGA graph can be interpreted as follows.

### Region I (500–610 °C, Δ*m*_1_ = 1.75%)

In this region, the initial weight loss is attributed to the onset of partial thermal decomposition of Sb_2_Se_3_. Literature reports indicate that Sb_2_Se_3_ begins to decompose in the 677–822 K (404–549 °C) range, forming volatile species such as Sb_4_ (g), SbSe (g), and Se_2_ (g).^20^ The observed mass loss in this temperature range suggests that selenium, due to its high vapor pressure, starts to volatilize even before complete decomposition occurs. This early selenium loss can contribute significantly to the minor weight reduction seen in this stage.

### Region II (610–775 °C, Δ*m*_2_ = 3.50%) and Region III (>775 °C, Sharp Weight Loss)

As the temperature increases, the decomposition of Sb_2_Se_3_ becomes more pronounced, leading to a more significant weight loss. Reaction 1 ^20^

$${\mathrm{Sb}}_{2}{\mathrm{Se}}_{3}\left(s\right)\to \frac{1}{4}{\mathrm{Sb}}_{4}\left(g\right)+\mathrm{SbSe}\left(g\right)+{\mathrm{Se}}_{2}\left(g\right)$$ Reaction 1

dominates in this region. Selenium volatilization intensifies, contributing to a major fraction of the mass loss. Alongside selenium loss, the formation and volatilization of SbSe and Sb_4_ also contribute to the observed decrease in mass.^21^ At temperatures above 775 °C, rapid weight loss may correspond to the final stages of selenium volatilization and potential evaporation of antimony. The relatively high residual mass (∼90%) observed near 800 °C suggests that not all volatile species have escaped the system. Incomplete decomposition or the presence of selenium-deficient phases (Sb_2_Se_3–*x*_) may also contribute to the observed residual mass.

The high thermal stability of Sb_2_Se_3_ observed up to 610 °C (this work) aligns well with previous studies reporting minimal structural degradation under elevated temperatures, which is critical for photovoltaic applications subjected to concentrated sunlight.^22,23^ Additionally, minimal weight loss below this temperature range supports the suitability of Sb_2_Se_3_ for thermoelectric devices, where thermal stress can be significant.^24^ The high-temperature stability of Sb_2_Se_3_ is particularly relevant for thermoelectric applications where operational temperatures can exceed 600 °C. In this context, Rahnamaye Aliabad et al.^24^ have reported an impressive figure of merit (ZT) of 1.12 at 523 °C (800 K) for n-type Sb_2_Se_3_ with a carrier concentration of *n* = 10^19^ cm^–3^. The negligible thermal degradation observed in our study supports the feasibility of operating Sb_2_Se_3_-based thermoelectric devices in similar temperature ranges. Taken together, these findings suggest that by appropriate doping and careful control of structural parameters, Sb_2_Se_3_ can achieve both high ZT values and sufficient thermal stability for reliable thermoelectric performance. It has also been reported in the literature that the thermoelectric performance of Sb_2_S_3–*x*_Se_*x*_ crystals increases with Se doping and the maximum ZT value is 6.87 × 10^–9^ at 390 K.^25^ The high thermal stability of Sb_2_Se_3_ reveals the potential for working in a wide temperature range in thermoelectric applications. The findings support the usability of Sb_2_Se_3_ as a thermoelectric material. The TGA results demonstrate that Sb_2_Se_3_ crystals exhibit thermal stability up to 600 °C. While such high temperatures are unlikely to be encountered during the operation of photovoltaic devices, this exceptional thermal stability is a clear advantage for material processing and long-term operational reliability. The ability of Sb_2_Se_3_ to maintain its structural and functional integrity under extreme thermal conditions makes it an ideal candidate for photovoltaic applications, where thermal stability contributes to enhanced durability and performance over extended periods. These findings further reinforce the suitability of Sb_2_Se_3_ as a robust absorber material in solar cell technology.

Considering both weight loss regions under the light of the first-order reaction case, activation energies (*E*_a_) of the corresponding decomposition processes were obtained by applying the Coats–Redfern expression given as^26,27^

$$\ln\left[\frac{-\ln\left(1-\alpha \right)}{T^{2}}\right]=\ln\left(\frac{AR}{\beta E_{a}}\right)-\frac{E_{a}}{RT}$$ 5

where *A* is the frequency factor, *R* = 8.314 J/mol·K is the gas constant, β = 20 K/min is the heating rate and, α is the conversion expressed as^28^

$$\alpha =\frac{m_{0}-m_{t}}{m_{0}-m_{f}}$$ 6

where *m*_0_, *m*_t_, and *m*_f_ are initial mass, mass at time *t*, and final mass, respectively. According to eq 6, the slope of the ln­[ – ln (1 – α)/*T* ^2^] vs 1/*T* plot is −*E*_*t*_/*R*. Figure 6 illustrates the linear fit analyses conducted for both decomposition phases, along with the activation energies obtained through the slope derived from the linear fit. The activation energy for the initial weight loss process was determined to be 121.8 kJ/mol. In contrast, the activation energy for the subsequent decomposition phase was found to be 57.2 kJ/mol. At this point, it will be valuable to compare these values with the thermal parameters of similar materials. The TGA plot of the Sb_2_S_3_ crystal showed a mass loss region between 664 and 900 °C.^29^ The analyses performed on this region showed that the relevant mass loss was related to the volatilization process and the corresponding activation energy was reported as 137.2 kJ/mol. To the best of our knowledge and research, there are no other reported activation energies for materials similar to the Sb_2_Se_3_ compound. Although there is no recorded activation energy, TGA plots reported on Sb_2_Se_3_ or similar compounds are available in the literature. As a result of TGA measurements made on Sb_2_Se_3_ powder, a weight loss starting around 423 °C and becoming sharper around 608 °C was observed.^17^ The TGA plot of the Bi_2_Te_3_ bulk compound presented mass loss starting from 420 °C and continued up to 600 °C.^30^ The total weight loss in this temperature range was around 1.38%. A 4.5% mass loss in Sb_2_S_3_ between 150 to 270 °C was reported in ref 31, and moss loss was increased starting from 500 °C, which was stated as a closer temperature value to the melting point of the compound.

**Figure 6:**
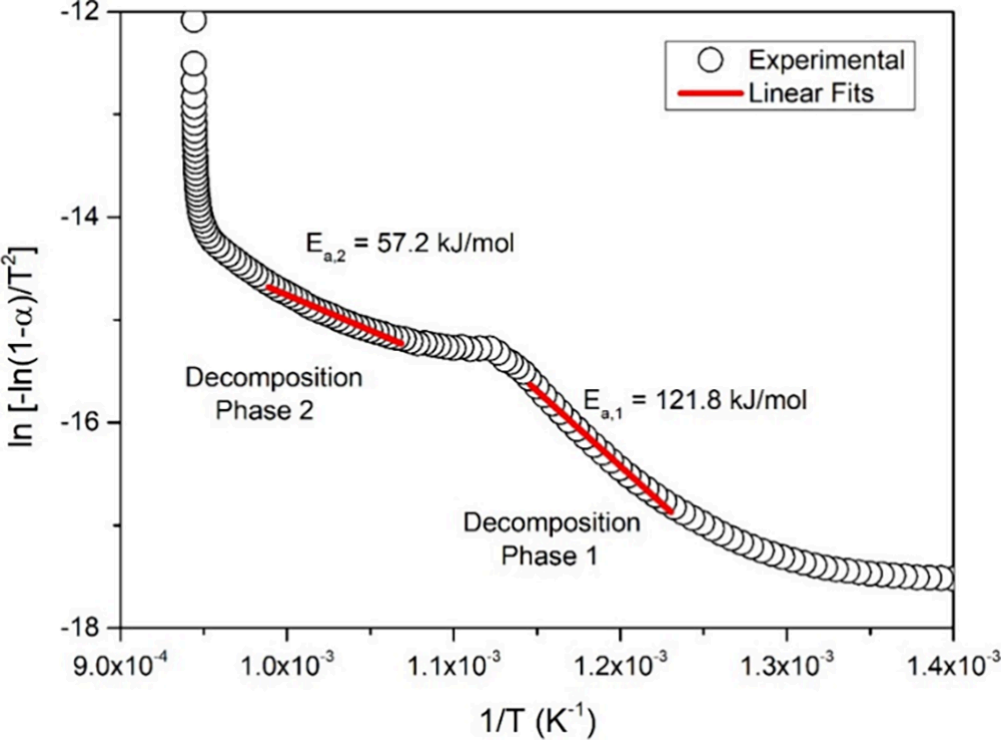
ln­[ – ln (1 – α)/*T* ^2^] vs 1/*T* plot.

Figure 7 shows the DTA plot of the Sb_2_Se_3_ crystal. The DTA curve exhibits an endothermic peak that begins around 599 °C and is completed near 630.6 °C. Based on the well-documented melting point of Sb_2_Se_3_ (∼611 °C) in the literature,^31^ this feature is attributed to the melting of the crystal rather than the release of absorbed water or volatile substances. This conclusion aligns with prior reports and provides further evidence of the thermal behavior of Sb_2_Se_3_. We see a general positive trend in the DTA signal between 200 and 600 °C. Changes in this region may indicate structural rearrangements or some thermal changes within the Sb_2_Se_3_ crystal. After 700 °C, the DTA signal drops rapidly downward. This may be associated with the decomposition of the Sb_2_Se_3_ crystal. The endothermic phase transition observed between 599.0 and 630.6 °C highlights a potential processing window for optimizing the crystalline quality of Sb_2_Se_3_ during device fabrication. Understanding and controlling this phase transition could enhance the performance and efficiency of Sb_2_Se_3_-based devices.^32^

**Figure 7:**
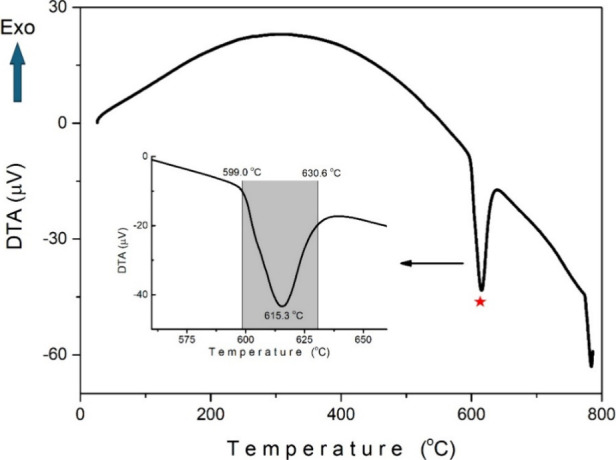
DTA plot of the Sb_2_Se_3_ crystal.

Figure 8 illustrates the experimental plots of DSC measurements conducted on the Sb_2_Se_3_ crystal at varying heating rates. DSC measurements were made between 50 and 450 °C. Since no peak was observed in the region below 300 °C, the graph was drawn between 300 and 450 °C for better understanding. The graph of the DSC measurement taken for a heating rate of 20 °C/min over the entire temperature range is given in the inset of Figure 8. It has been observed that the peak minimum points shift to higher temperatures with an increase in the heating rate. The recorded peak minimum points were 377.1, 408.0, and 417.9 °C for heating rates of 5, 15, and 20 °C/min, respectively. The primary factors responsible for the observed shift in peak values toward higher temperatures with increasing heating rates can be attributed to the following:^33,34^ (i) *Thermal lag* occurs when higher heating rates impede the ability of the sample to achieve equilibrium with the temperature increase, resulting in a delay between the sample and its surroundings. Consequently, the sample requires higher temperatures to achieve the same thermal energy level observed at lower heating rates. (ii) The phenomenon of *kinetic effects* arises from the rearrangement of molecular or atomic structures that occurs throughout the phase transitions. With the application of higher heating rates, the rate of these structural changes is accelerated, driven by the rapid temperature rise. Consequently, phase transitions occur at higher temperatures in order to surpass the activation energy barriers associated with the transitions. (iii) *Heat transfer effects* can be more pronounced when higher heating rates are used, and this can place restrictions on the rate at which heat is transferred to the sample. This limitation can cause temperature gradients within the sample, which can cause the observed peak temperatures to shift upward.

**Figure 8:**
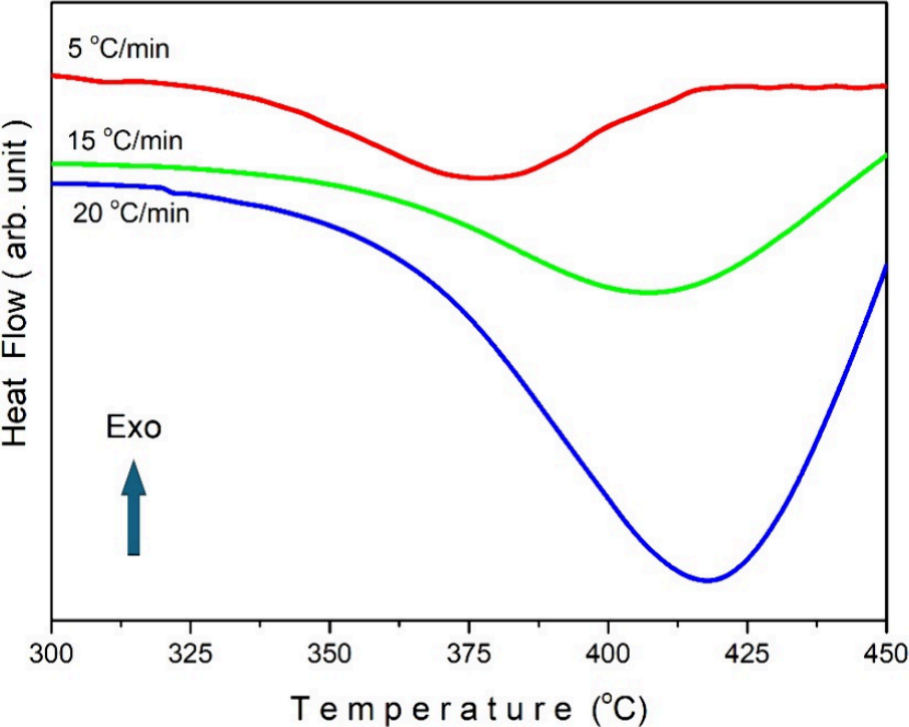
DSC plots of the Sb_2_Se_3_ crystal for various heating rates.

The observed endothermic process may be related to the crystallization. Although our studied material has a crystalline structure, it is possible that new crystal grains or crystal growth may occur within this crystal structure. The following Kissenger expression was utilized to determine the activation energy of corresponding crystallization process^35^

$$\ln(\beta /T_{m}^{2})=-(E_{a}/RT_{m})+C$$ 7

where *T*_m_ and *C* are the peak minimum temperature and a constant, respectively. Equation 8 implies that the slope of the ln­(β/*T*_m_^2^) vs 1/*T*_m_ graph is equal to −*E*_a_/*R*. Figure 9 shows the corresponding graph and linear fit line. The activation energy obtained using the slope of the linear fit line was found to be 117.2 kJ/mol.

**Figure 9:**
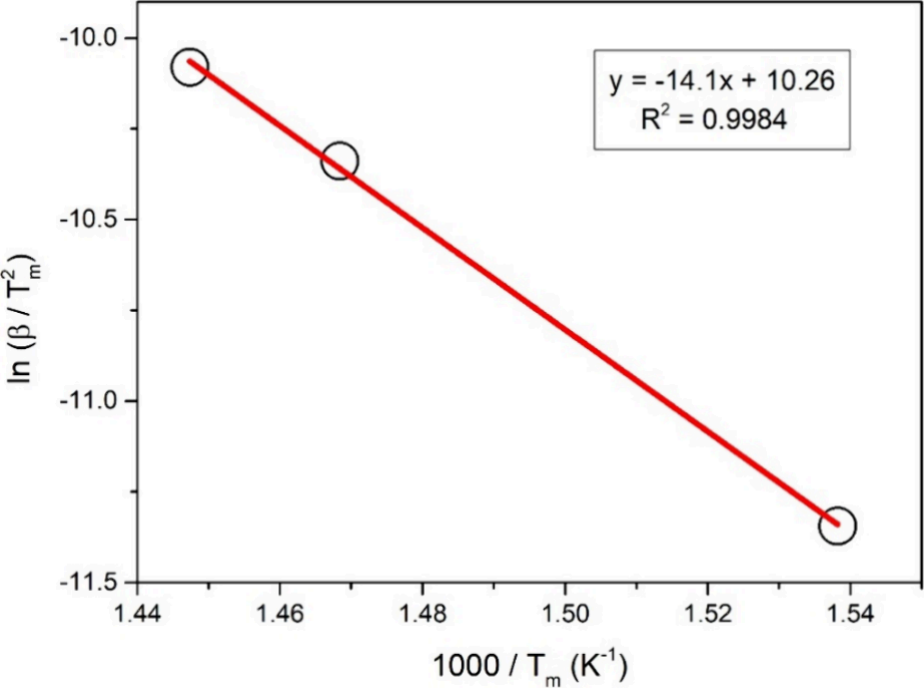
ln­(β/*T*_m_^2^) vs 1000/*T*_m_ plot for Kissenger analysis.

The DSC plot can be utilized to get the lifetime (degradation time) of a material. Lifetime is the term that expresses how long a material can remain at any temperature without decomposition and is calculated with the help of the following equation^36^

$$\tau_{i}=\frac{\int_{T_{0}}^{T_{i}}\exp(-\Delta E/RT)dT}{\beta \exp\left(-\frac{\Delta E_{i}}{R(T_{c}+273)}\right)}$$ 8

The temperature dependence of the lifetimes obtained as a result of the calculations made for 50 °C intervals between 50 and 450 °C is plotted in Figure 10. As expected, it was observed that the lifetime decreased with increasing temperature in the Sb_2_Se_3_ compound. The lifetime analysis presented in Figure 10 provides insight into the long-term stability of Sb_2_Se_3_ at different temperatures. While the material maintains its chemical integrity at high temperatures, the observed decrease in lifetime suggests that kinetic factors may influence its prolonged stability. These results show that while Sb_2_Se_3_ is thermally stable, prolonged exposure to high temperatures may lead to material degradation over time, depending on the operating conditions. The temperature vs lifetime relationship is more exponential than linear. Considering this situation, the log­(τ) vs 1000/*T* graph was plotted in order to show the relationship between these two parameters.^37^ When this plot shown in the inset of Figure 10 was fitted linearly, the slope was found to be 7.569 day·K and the intercept was found to be −12.07.

**Figure 10:**
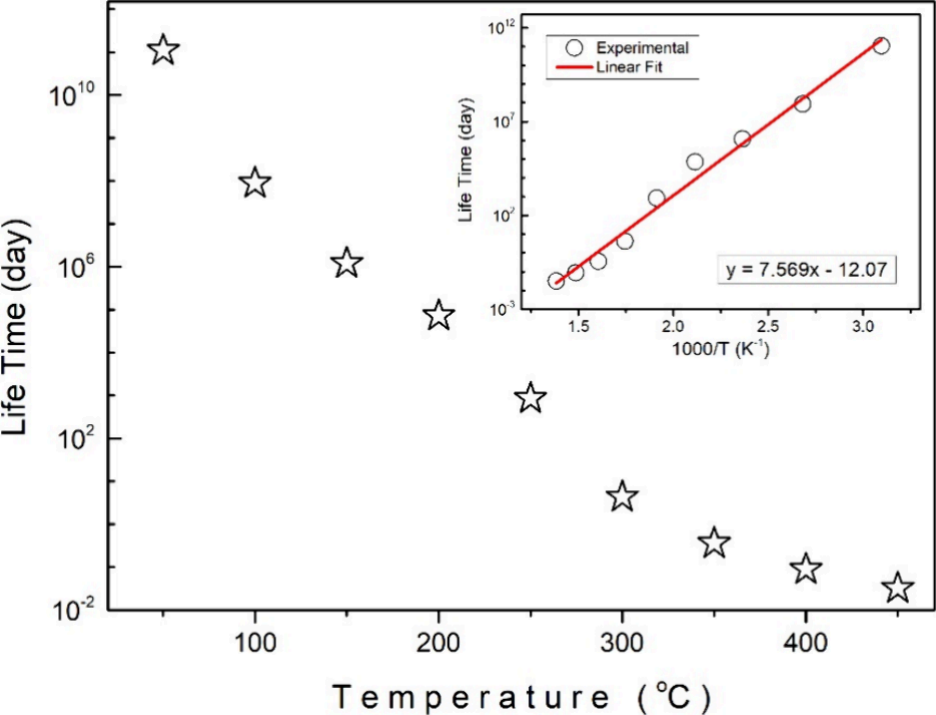
Temperature-dependent lifetime plot.

## 4 Conclusions

In the present work, structural and thermal properties of the Sb_2_Se_3_ crystal were reported. The XRD pattern indicated 20 peaks associated with orthorhombic crystal structure. Analysis of the XRD pattern resulted in lattice parameters of *a* = 11.770 Å, *b* = 3.9757 Å, and *c* = 11.6327 Å. The strain and crystalline size of the compound were obtained as −1.8 × 10^–3^ and 31.7 nm, respectively. The TGA plot indicated that there is no weight loss up to 500 °C and there exist two weight loss zones above this temperature. Up to 610 °C, the weight loss was observed as %1.75 while the weight loss was %3.50 in the temperature region of 610–775 °C. In the region above 775 °C, it was observed that the weight decreased significantly with the increase in temperature. The fact that the weight loss in the Sb_2_Se_3_ compound is not significant up to 610 °C shows that the thermal resistance of the compound is up to this temperature. The activation energies of the decomposition processes in the weight loss zones were found to be 121.8 and 57.2 kJ/mol using the Coats–Redfern expression. The DTA plot presented an endothermic phase transition taking place between 599.0 and 630.6 °C. As a result of DSC measurements performed at different heating rates, it was observed that the peak minimum point shifted from 377.1 to 417.9 °C. The activation energy of the endothermic process associated with this peak was found to be 117.2 kJ/mol using the Kissenger equation. This study provides an in-depth study of the thermal properties of the Sb_2_Se_3_ crystal, demonstrating the material’s potential for use in environments requiring high-temperature resistance in solar cells and other photovoltaic and optoelectronic applications.

## Data Availability

The data that support the findings of this study are available from the corresponding author upon reasonable request. The data are not publicly available due to intellectual property protection related to future commercial applications.
